# Three-dimensional-printed porous prosthesis for the joint-sparing reconstruction of the proximal humeral tumorous defect

**DOI:** 10.3389/fbioe.2022.1098973

**Published:** 2023-01-12

**Authors:** Yuqi Zhang, Minxun Lu, Xin Hu, Zhuangzhuang Li, Jie Wang, Taojun Gong, Yong Zhou, Li Luo, Li Min, Chongqi Tu

**Affiliations:** ^1^ Department of Orthopedics, Orthopedic Research Institute, West China Hospital, Sichuan University, Chengdu, Sichuan, China; ^2^ Model Worker and Craftsman Talent Innovation Workshop of Sichuan province, Chengdu, Sichuan, China

**Keywords:** 3D printed, arthroplasty, prosthesis, intercalary prosthesis, proximal humerus

## Abstract

**Background:** Tumorous bone defect reconstructions of the proximal humerus with joint sparing is a challenge. Numerous reconstruction methods have been proposed but the proximal residual humerus is commonly sacrificed because of its extremely short length. To preserve the proximal humerus and improve clinical outcomes, we designed a three-dimensional (3D) printed uncemented prosthesis with a porous structure to treat tumorous bone defects of the proximal humerus.

**Methods:** Our analysis included seven patients treated between March 2018 and July 2019. A 3D model was established, and related data were obtained, including the diameter of the humeral head, the resection length, and the residual length. A prosthesis was designed and fabricated based on these data. Functional and oncologic outcomes were recorded, and complications and osseointegration were evaluated.

**Results:** The mean age of the patients was 20.3 years, and the median follow-up period was 26 months. The lengths of the residual proximal humerus were 17.9 mm on average. All the patients had preserved humeral heads and most of the rotator cuff was intact. The average postoperative range of motion (ROM) of the affected shoulder was 83.8°; flexion was 82.5°, extension was 43.8°, and adduction was 16.3°. The average Musculoskeletal Tumor Society score (MSTS) was 94.3%. Good osseointegration was observed on the interface between the bone and prosthesis.

**Conclusion:** A 3D printed porous prosthesis with cone-like structures successfully achieved joint-sparing reconstruction of proximal humeral tumorous defects with satisfying functional outcomes. The preservation of the rotator cuff and humeral head plays an essential role in the function of the shoulder joint.

## 1 Introduction

The metaphysis of the proximal humerus is the most commonly affected site for primary malignant bone tumors (Bielack et al., 2002; Arndt et al., 2012). Although segmental resection with a safe margin has been widely accepted as the standard treatment for malignant bone tumors (Potter et al., 2009), joint preservation is still demanding due to the extremely short axial length of the residual proximal humerus (Liu et al., 2014).

There are currently some approaches available for the repair of tumorous defects involving the metaphysis, such as autograft, allograft, and prostheses (Ruggieri et al., 2011; Wieser et al., 2013; King et al., 2016; Maclean et al., 2017; Rafalla and Abdullah, 2017; Nota et al., 2018; Chauhan et al., 2019). Autologous fibula graft has been widely applied in clinical settings because of its excellent biocompatibility and osteoinductivity (Li et al., 2012; Pilge et al., 2018). However, the interface may not integrate well due to the severe mismatch between the fibula head and the remaining proximal humerus, and subsequent bone absorption and fracture frequently occur (Ceruso et al., 2001). Therefore, an allograft with various options for appropriate size and shape could provide an ideal interface contact, but unexpected immunological rejection and disease transmission are still major concerns (Gupta et al., 2017). As a result, prostheses seem to be one of the most acceptable choices for the reconstruction of segmental bone defects in the proximal humerus (Damron et al., 2008).

Hemiarthroplasty, total arthroplasty, and intercalary prosthesis replacements are all reasonable options for the reconstruction of proximal humeral tumorous defects. As for defects involving the metaphysis, hemiarthroplasty or total arthroplasty would inevitably sacrifice the humeral head, which could have been preserved. Compared with hemiarthroplasty or total arthroplasty, an intercalary prosthesis could not only preserve the humeral head anatomically to retain shoulder function but also provide early stability and rapid function recovery (Yoshida et al., 2010; Panagopoulos et al., 2017). However, current intercalary prostheses are cemented and fixed by an intramedullary stem, which requires at least 3 cm of bone to maintain acceptable stability (McGrath et al., 2011). Moreover, plates and screws are still required for extra fixation due to the lack of osteoinductive activity and bone ingrowth ability (Zekry et al., 2019; Zheng et al., 2019). Additionally, for a shorter proximal humerus (length < 3 cm), reconstruction with joint sparing cannot be achieved by intercalary prosthesis.

Recently, it has been accepted that the porous structure could significantly improve the integration ability of a prosthesis (Batta et al., 2014). In our previous clinical evaluation of three-dimensionally (3D)-printed porous intercalary prostheses, excellent interfacial integration was observed, even with a residual bone length shorter than .7 cm (Lu et al., 2018; Zhao et al., 2020). In this study, we aimed to design and apply a new 3D printed uncemented prosthesis with special features and evaluate its feasibility for the treatment of proximal humeral defects. The detailed design and features of the prosthesis, surgical techniques, and early-term clinical outcomes are presented and analyzed.

## 2 Materials and methods

### 2.1 Patients

**FIGURE 1:**
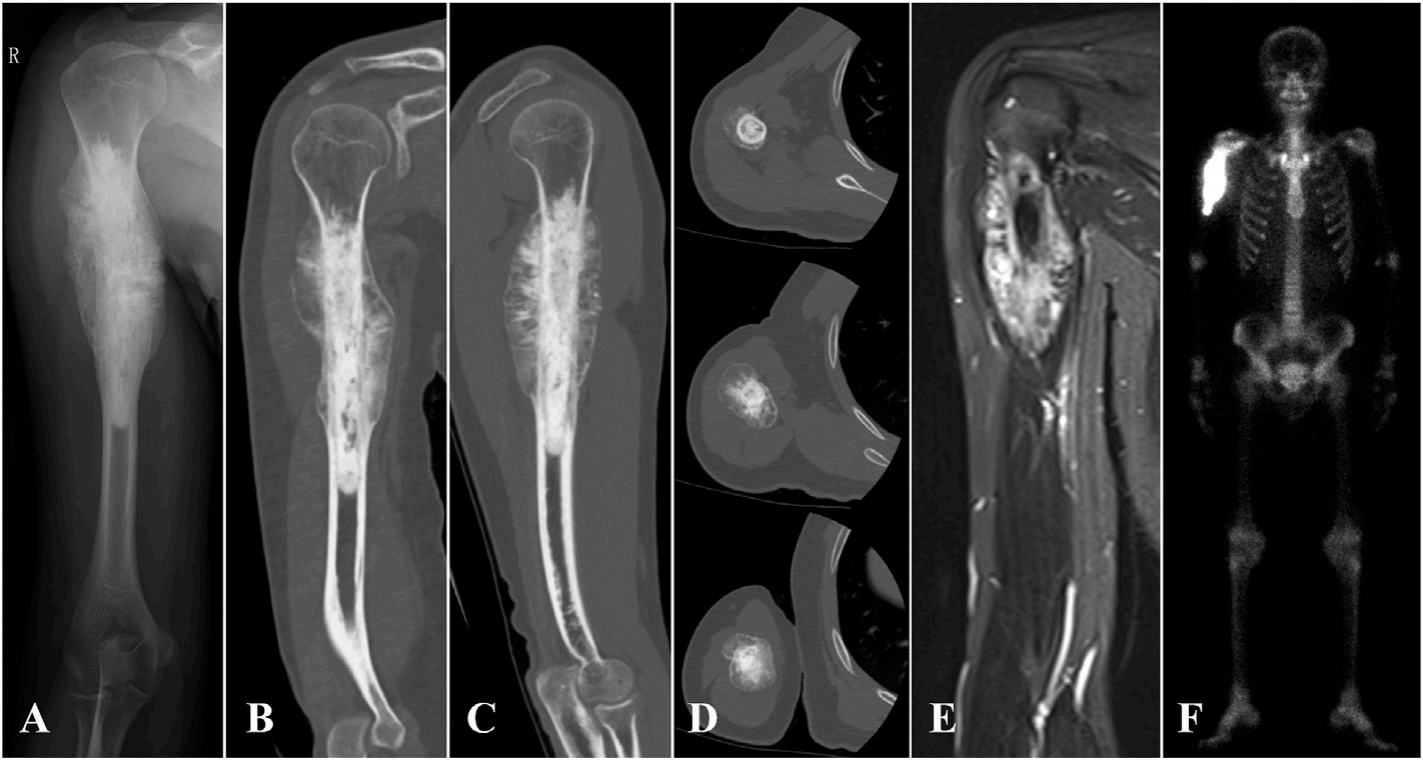
**(A)** X-ray, **(B–D)** 3D CT, **(E)** MRI, and **(F)** SPE/CT of case 1 with proximal humerus osteosarcoma are shown.

Between March 2018 and July 2019, seven patients (two females and five males) with humeral malignant tumors received 3D printed uncemented prosthesis reconstructions in our institution. The average age was 19.25 years (range, 16–24 years). All the patients received preoperative radiographic assessments, including x-rays, 3D computed tomography (CT) scans (Philips Brilliance 64 Slice, thickness: .4 mm), magnetic resonance imaging (MRI) scans, and bone scans (SPECT) or positron emission tomography/computerized tomography (PET/CT) scans (Figure 1). A preoperative biopsy was performed for all patients. An Enneking surgical staging system was used to evaluate the surgical stage (Enneking, 1986). Tumor locations were classified with reference to the epiphyseal plate proposed by Kumta et al. (Kumta et al., 1999). Neoadjuvant chemotherapy was performed for patients with high-grade sarcoma according to the NCCN guidelines for bone cancer. The detailed characteristics of the patients are summarized in Table 1.

**TABLE 1:** Patient characteristics.

Case	Age	Gender	Follow up (months)	Stage	Classification	Pathological type
1	17	Male	33	IIB	II	Osteosarcoma
2	20	Male	25	IB	III	Chondrosarcoma
3	24	Female	29	IIB	III	Osteosarcoma
4	16	Male	18	IIB	II	Osteosarcoma
5	16	Male	21	IIB	II	Osteosarcoma
6	19	Female	30	IB	II	Chondrosarcoma
7	30	Male	25	IIB	III	Chondrosarcoma

This study was performed in accordance with the Declaration of Helsinki as revised in 2008 and was approved by the Ethics Committee of the West China Hospital. All patients signed an informed consent form before surgery and provided consent to publish and report individual clinical data.

### 2.2 Anatomical data measurement

**FIGURE 2:**
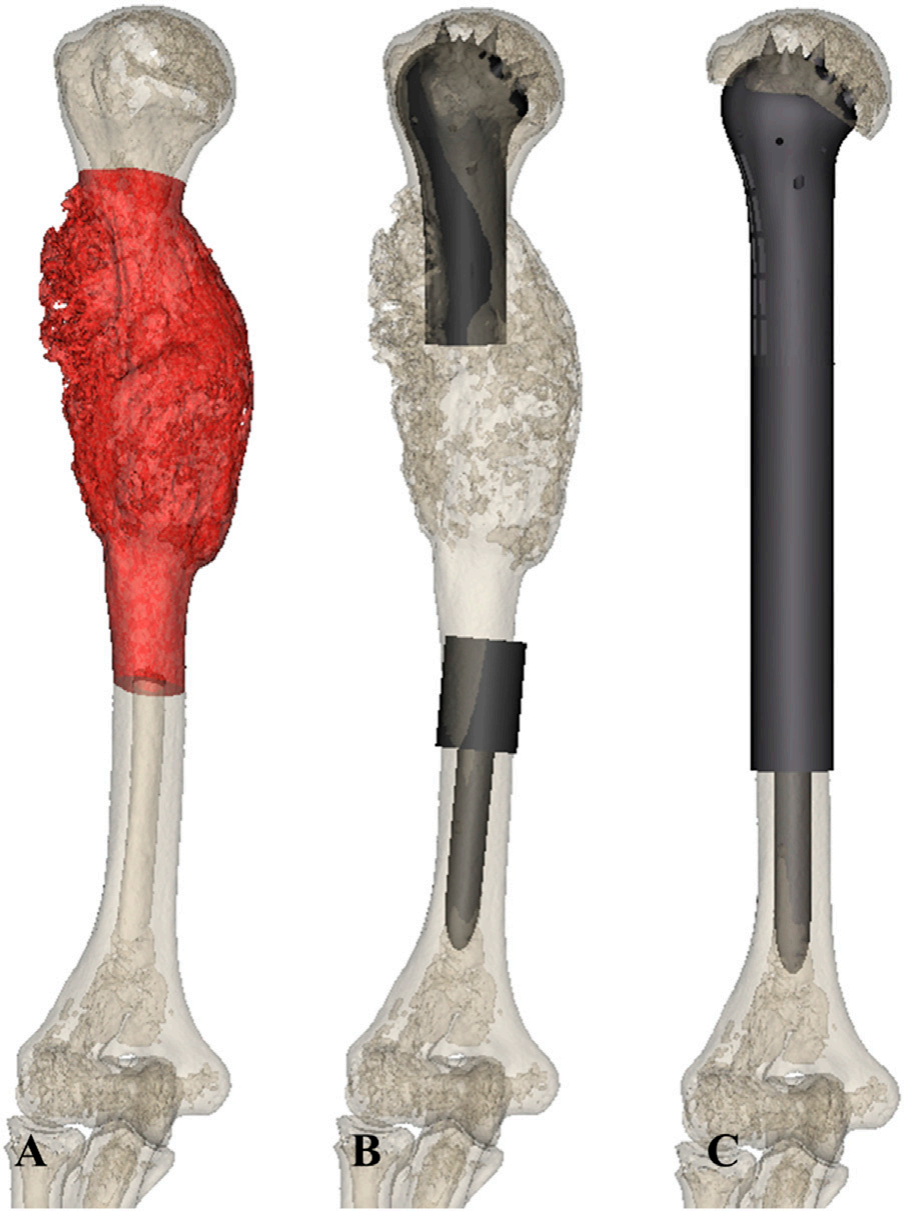
**(A)** The model of the humerus with tumor was established using 3D CT and MRI. **(B,C)** Prosthesis models **(B,C)** were designed based on the anatomy data.

The 3D CT data of patients were imported to Mimics V20.0 software (Materialise Corp., Leuven, Belgium) to build virtual 3D models of the tumor and bone. The tumor edge was determined using the combination of x-ray, MRI, and SPECT. Anatomical data, including the diameter of the humeral head, the proximal and distal osteotomy location, the resection length, the length of the residual humerus, and the diameters of the intramedullary cavity, were obtained. The curative margin was subsequently obtained to determine the tumor resection and residual bone parts, and an operation simulation was performed using Geomagic Wrap software (Geomagic inc., Morrisville, NC) (Figure 2A).

### 2.3 Prosthesis design and fabrication

All prostheses were designed by our clinical team according to the anatomical data, and were fabricated by Chunli Co., Ltd., Tongzhou, Beijing, China. The prostheses consisted of a head, shaft, and stem. A hemisphere-like structure was selected for the design of the head shape, and the size of the head was customized in all patients as per the host humeral head. In addition, specific features, including suture holes and cone-like structures, were added to enhance the initial stability of the bone-implant interface. In detail, a solid core porous shell complicated structure concept was applied to design the prosthesis head. The thickness of the porous shell layer was 3–4 mm. Furthermore, 600-µm pores with 70% porosity were suggested for the setting of the porous shell (Karageorgiou and Kaplan, 2005; Palmquist et al., 2013; Hara et al., 2016; Shah et al., 2016; Wang et al., 2019). The shaft length depended on the bone defect length of the patient (Figures 2B, C).

**FIGURE 3:**
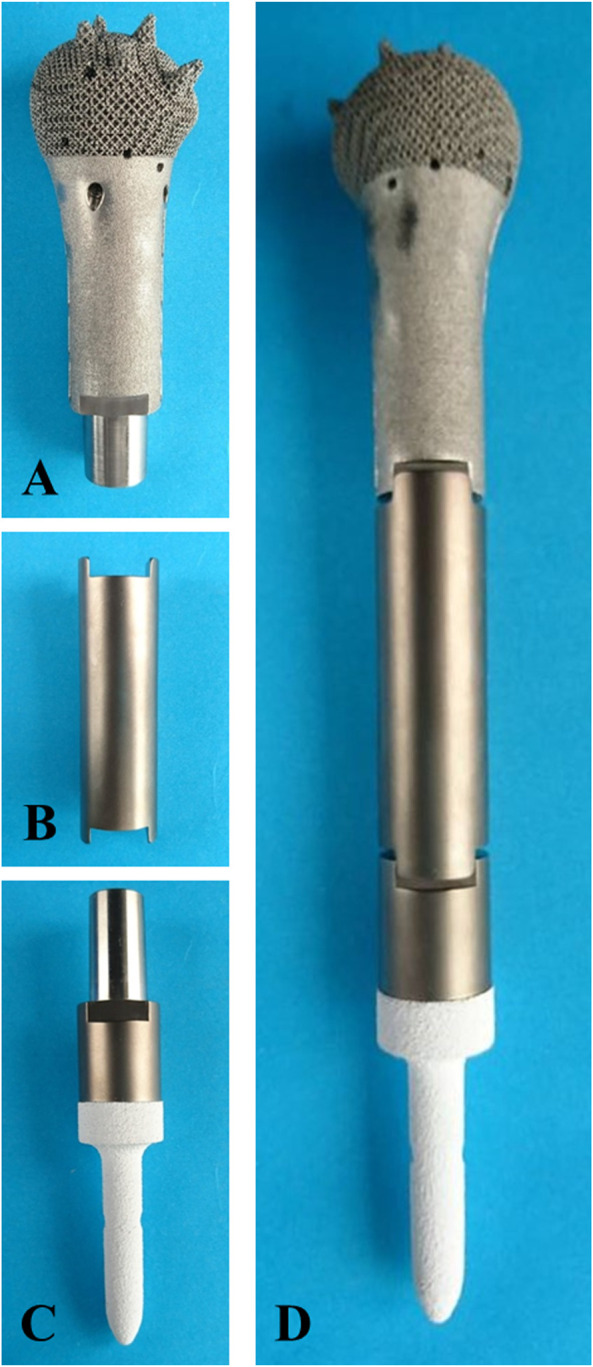
Fabricated prosthesis pictures. **(A)** Prosthesis head. **(B)** Prosthesis shaft. **(C)** prosthesis stem. **(D)** Assembled prosthesis.

The prosthesis was made of titanium alloy (Ti6Al4V powder, Chunlizhengda Corp., Beijing, China) and was fabricated using the electron beam melting technique (ARCAM Q10 plus, Mölndal, Sweden) with the powder bed fusion technique. The metal powder was placed in a vacuum and fused by heat from an electron beam. The components were then fabricated as per the previously designed model by the continuous addition of pre-alloyed powder layers. The plastic patient-specific instruments and trial models were fabricated *via* stereo lithography apparatus techniques (UnionTech Lite 450HD, Shanghai, China) (Figure 3).

### 2.4 Surgical techniques

**FIGURE 4:**
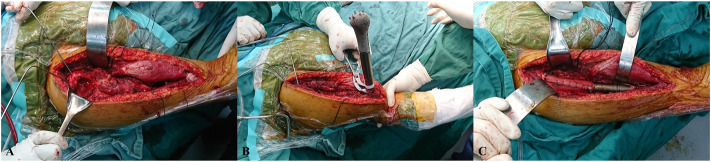
Intraoperative pictures. **(A)** The remaining proximal humerus was trimmed after segmental resection. **(B)** The prosthesis stem was press-fit after assembly. **(C)** The prosthesis was implanted, and muscles were reconstructed using Marlex mesh.

All patients were placed in a supine position. Through an anterior longitudinal humeral incision, the radial nerve was exposed and well protected. The preservation of the rotator cuff insertions was performed before the segmental resection. The osteotomy was performed with patient-specific instruments, in reference to the greater tubercle of the humerus. The remaining proximal humerus was trimmed, while reaming was performed to press-fit the prosthesis. The bone marrow from the reamed canal and trabecular bone trimmings were collected for subsequent autograft (Figure 4A).

The prosthesis was implanted after autografting. Extra fixation depended on the intraoperative initial stability of the prosthesis. When the prosthesis was appropriately in place, axial compression was carried out to press the cone-like structure into the proximal cancellous bone. The remaining proximal humeral cortex was sutured to the prosthesis head with the rivet lines, and the sutured cortex needed to be sufficiently stable to prevent avulsion (Figure 4B).

The residual muscles were anatomically relocated to the prosthesis surface with rivet lines but without knotting. The muscles posterior and medial to the humerus were sutured first, followed by the muscles anterior and lateral to the humerus. The knots to suture the rotator cuff were finished together to balance perishoulder muscle tension. The deltoid, pectoralis major, and pectoralis minor, which are important for shoulder ROM, were finally reconstructed, and soft tissue coverage was achieved. Intraoperative time and blood loss were recorded (Figure 4C).

### 2.5 Postoperative treatment and follow up

The affected limbs of patients were immobilized at 80° of abduction and 60° of flexion for 4 weeks. Passive movements were allowed after week 4, and patients were gradually transited to active movement at week 6. The exact time of lifting and exercise depended on the degree of osseointegration. Postoperative chemotherapy was started 2 weeks after surgery.

All patients underwent evaluations, including monthly physical examinations and radiography, during the first 3 months postoperatively and every 3 months thereafter. The absence of periprosthetic radiolucency or the observation of bone bridging, spot welding, and neocortex formation between the trabecular structures and the implant surface on x-rays or Tomosynthesis-Shimadzu Metal Artefact Reduction Technology (T-SMART) was considered good osseointegration. Chest CT scans were used to evaluate lung metastasis every 3 months. Functional outcomes were assessed using the Musculoskeletal Tumor Society score (MSTS), ROM of the glenohumeral joint was recorded, and complication rates were assessed.

### 2.6 Statistical analysis

Statistical analyses were performed using IBM SPSS Statistics software, version 22 (IBM SPSS, Armonk, NY, United States). Continuous data are represented as mean ± standard deviation. Student’s *t*-test was used to compare continuous variables. *p* < .05 was considered statistically significant.

## 3 Results

Detailed measurement data are summarized in Table 2. The mean diameter of the humeral head was 42.4 ± 2.0 mm. The mean resection length was 130.5 ± 47.5 mm, and the mean lengths of the residual proximal humerus and residual distal humerus were 17.9 ± 1.3 and 155.5 ± 50.2 mm, respectively.

**TABLE 2:** Anatomy data.

Case	Diameter of the head	Proximal OP distance^a^	Distal OP distance	Resection length	Length of the RPH	Length of the RDH	Diameter of the RDH intramedullary cavity
1	42.24	19.82	201.67	181.85	19.82	103.66	10.29
2	45.93	16.59	92.21	75.62	16.59	237.31	11.94
3	43.33	17.85	169.35	151.5	17.85	142.42	11.01
4	40.23	16.22	101.38	85.16	16.22	193.64	10.03
5	40.11	19.01	180.5	161.49	19.01	118.56	11.21
6	42.56	18.55	100.5	81.95	18.55	182.34	11.66
7	42.65	17.21	193.23	176.02	17.21	110.19	9.86
Mean	42.44	17.89	148.41	130.51	17.89	155.45	10.86

^a^ Osteotomy plane distance indicates the distance from the osteotomy plane to the proximal end of the humerus.

OP, osteotomy plane; RPH, residual proximal humerus; RDH, residual distal humerus.

Detailed prostheses data are summarized in Table 3. The mean diameter of the prosthesis head was 34.7 ± 1.3 mm. According to different resection lengths, the mean length of the prosthesis was 178.6 ± 50.1 mm. Based on the residual length and the diameter of the intramedullary cavity, the mean length and diameter of the prosthesis stem were 48.6 ± 3.8 mm and 10.7 ± .8 mm, respectively.

**TABLE 3:** Prosthesis data (mm).

Case	Prosthesis length	Length of the stem	Diameter of the stem	Diameter of the head	Diameter of the body
1	240	55	10	35	20
2	120	45	12	36	22
3	200	50	11	36	20
4	130	45	10	33	18
5	210	50	11	33	20
6	130	50	11	35	22
7	220	45	10	35	20
Mean	178.57	48.57	10.71	34.71	20.29

Surgeries took 3.3 ± .7 h and the mean volume of intraoperative hemorrhage was 225.7 ± 59.1 ml. The entire supraspinatus and most of the infraspinatus were preserved in every case. The teres minor was rarely preserved, and part of the subscapularis was preserved.

**FIGURE 5:**
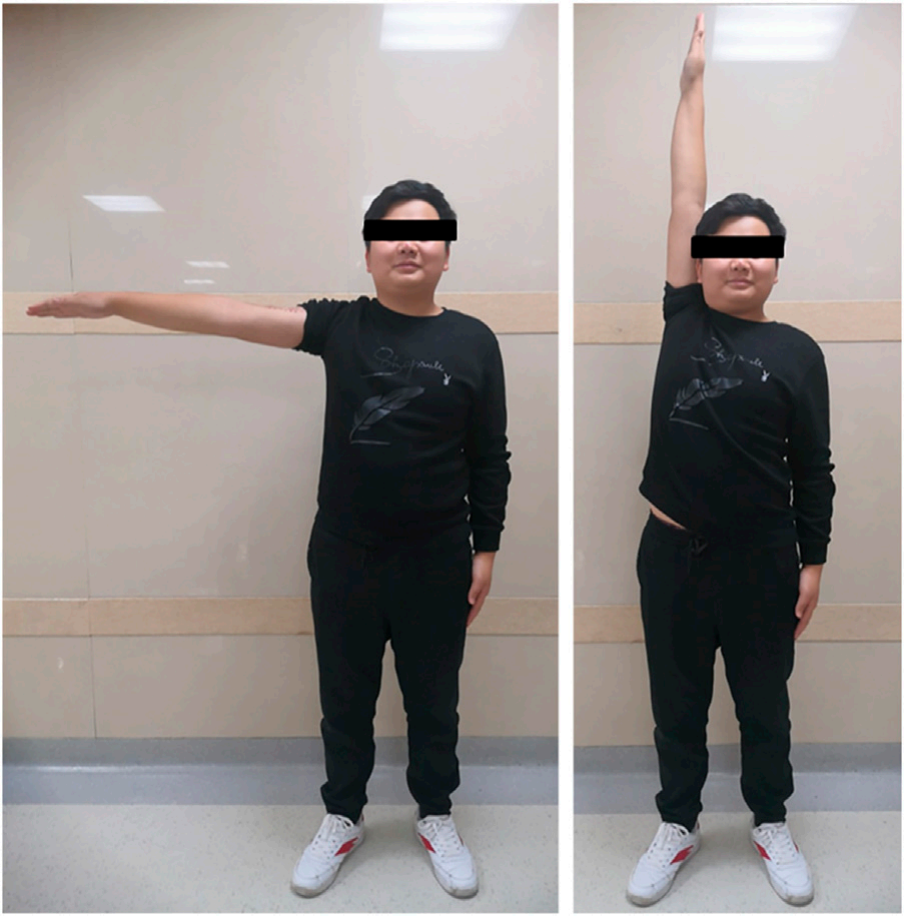
The shoulder abduction of case 1 was normal 6 months after surgery.

**FIGURE 6:**
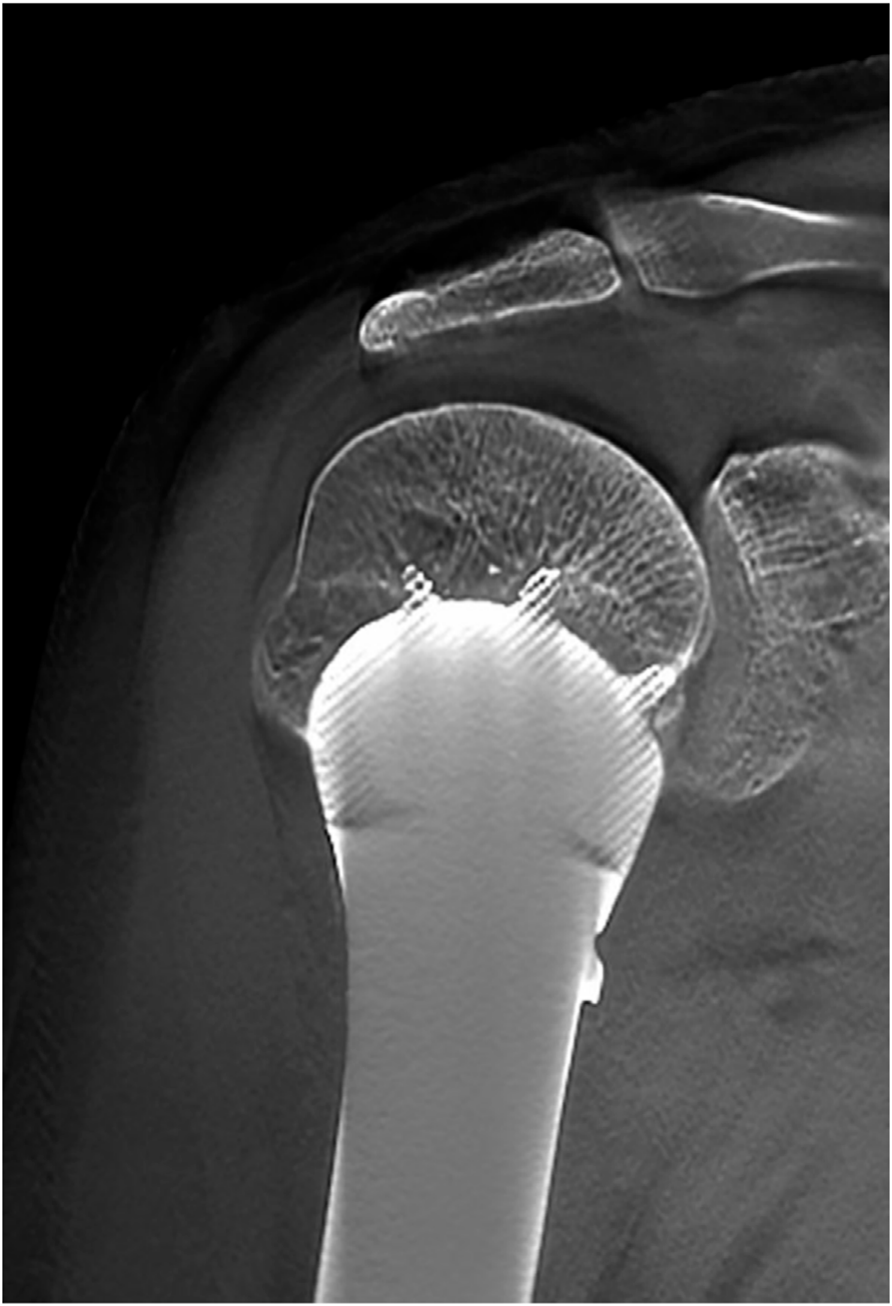
T-SMART showed preliminary osseointegration 6 months after surgery.

The mean follow-up period was 26 months (range, 18–33 months). The average MSTS score was 94.3%, which increased with statistical difference (*p* < .05). Average abduction was 83.8°, flexion was 82.5°, extension was 43.8°, and adduction was 16.3° in this series (Figure 5). No aseptic loosening, breakage, dislocation, and infection of prostheses were found until the last follow up. No local recurrence and distant metastasis were observed in all cases. One patient had radial nerve palsy, which recovered completely 5 weeks after surgery. The absence of radiolucency between the prosthesis and the bone was observed with T-SMART 6 months postoperatively (Figure 6). Intraoperative data and oncologic and functional outcomes are summarized in Table 4.

**TABLE 4:** Intraoperative data and oncologic and functional outcomes.

Case	Surgery time (h)	Intraoperative hemorrhage (ml)	Tissue preservation				Closest margin (mm)	Local recurrence	Limb shortening (mm)	Complication	MSTS score (%)
			Musculus infraspinatus	Musculus supraspinatus	Musculus teres minor	Musculus subscapularis					
1	2.1	150	Most	All	None	Partial	10.0	No	3.25	—	93
2	3.5	230	Most	All	Little	Partial	10.0	No	5.53	Radial nerve palsy	90
3	2.7	200	Most	All	Little	Partial	9.0	No	−2.67	—	97
4	4.2	340	Most	All	Little	Partial	9.0	No	3.60	—	93
5	3.0	200	Most	All	Little	Partial	9.5	No	−1.49	—	97
6	3.1	250	Most	All	Little	Partial	9.0	No	−1.95	—	97
7	4.0	210	Most	All	Little	Partial	10.0	No	−1.02	—	93
Mean	3.23	225.71	—	—	—	—	9.5	—	.75	—	94.29

## 4 Discussion

Tumorous defect reconstructions with shoulder joint preservation involving the metaphysis are challenging due to the limited surgical techniques and prosthesis designs. Our 3D printed porous prosthesis with cone-like structures successfully achieved joint-sparing reconstruction of proximal humeral tumorous defects with satisfying functional outcomes.

Prosthetic reconstruction is probably the most widely used method because of its availability, low complication rates, and acceptable functional results compared with other approaches (Ceruso et al., 2001; Gupta et al., 2017). The resection length and residual bone are important parameters for the application of an intercalary prosthesis. Benevenia et al. suggested that an intercalary prosthesis is a good reconstruction method for humeral defects when residual bone length is ≥ 4 cm (Benevenia et al., 2016). However, the length of intramedullary fixation severely restricted the application of the prosthesis. Abudu et al. (1996) showed that an intercalary prosthesis has a high risk of early loosening when the intramedullary fixation length is < 5 cm. When the fixation length is < 4 cm, a previous study advocated for extracortical plates to enhance fixation (Sewell et al., 2011). When the residual bone length is < 3 cm, or even 2 cm, the remaining humeral head has to be sacrificed for arthroplasty, which leads to potentially increased instability and a permanent reduction in limb function (Hardes et al., 2013).

In our study, the newly porous prosthesis successfully reconstructed the humeral defect and preserved the shoulder joint. Average ROM was 83.75° for abduction, 82.5° for flexion, 43.75° for extension, and 16.25° for adduction. The functional results showed an average MSTS score of 94.29%, which is better than those of other series (Wittig et al., 2002; Li et al., 2012; Zheng et al., 2019). In all cases, periprosthetic radiolucency disappeared 6 months postoperatively, and bone ingrowth could be observed with T-SMART.

Good osseointegration induced by uncemented fixation is essential for the long-term survival of the prosthesis with joint preservation. By contrast, cemented fixation failed to achieve joint-preserving reconstruction in cases of residual bone length of < 2 cm, and certainly cannot achieve interfacial integration, which inevitably results in prosthesis loosening or dislocation. McGrath et al. reported on 13 patients who underwent cemented intercalary prosthetic reconstruction (the shortest intramedullary stem = 3 cm) for malignant bone disease of the humerus (McGrath et al., 2011). Aseptic loosening occurred in four cases, and two patients presented with periprosthetic fractures. The overall complication rate related to the prosthesis was 31%. To promote osseointegration, the structure of the prosthesis was optimized. Previous studies showed that the porous structures (pore size of 300–800 µm and porosity of 70%) at the interface could enhance bone ingrowth (Karageorgiou and Kaplan, 2005; Palmquist et al., 2013; Hara et al., 2016; Shah et al., 2016; Wang et al., 2019). So, porous structures with a porosity of 70% and pore size of 600 µm were applied in the shell layer of the prosthesis head in our study.

However, osseointegration could be affected by micromotion through the formation of fibrous tissues and the induction of bone resorption (Pilliar et al., 1986). To reduce micromotion, we designed cone-like structures with uniform distribution on the prosthesis head. The advantages of the cone design are as follows. First, the design increases the contact surface and friction to reduce micromotion. Second, axial pressure makes the cones anchor into the spongy bone, which improves the initial rotational stability. Third, the contact of the cancellous bone and cones increases the shear force of the interface to avoid possible relative displacement. Furthermore, the tight sutures of the humeral head to the prosthesis provide axial stability to prevent the separation of the bone and the prosthesis. Moreover, all patients were restricted to passive movements of the involved shoulder by limb immobilization within the first 4 weeks, during which time bone ingrowth occurred. The optimization of the porous shell and the control of micromotion laid the foundation for subsequent functional rehabilitation. Comparatively, cemented intramedullary fixation relies on the adhesive properties of cement to provide initial stability. Zhao et al. (2020) suggested that high rotational stress and traction play a role in the early loosening of cemented fixation and recommended adding a preventive additional extracortical plate to share the partial stress of the intramedullary fixation. However, extracortical plate implantation increases the risk of prosthesis related complications, such as infection and rupture.

There were no cases of subluxation or dislocation in our study. Patients could even complete circumduction movements in the sagittal plane (Supplementary Video S1). Dynamic x-rays showed smooth movement of the shoulder joint (Supplementary Video S2). By comparison, patients have been reported to present with decreased shoulder abduction after resection of the proximal humerus, even after undergoing arthroplasty or bone graft (Wittig et al., 2002). In Dubina’s review, 84 patients from eight studies of shoulder arthroplasty were analyzed. The mean MSTS score was 70%, and 26% patients presented with mechanical failure. The favorable result of our study depends not only on the prosthesis design rationale but also on important anatomic structure preservation and precise surgical techniques. First, the preservation of the shoulder joint and rotator cuff directly resulted in good function of the shoulder joint. Although direct attachment of the residual rotator cuff to the allograft or prosthesis was performed, some proximal subluxation inevitably occurs because of the improper reconstruction of the rotator cuff (Ayoub et al., 1999; Black et al., 2007). Joint preservation reduced the difficulty of muscle reconstruction and the damage to the muscle insertions, which enhanced postoperative recovery. Second, the preservation of the articular capsule and rotator cuff maintained the passive tension of the joint. When the shoulder joint is in motion, passive tension in the rotator cuff provides compressive stress between the articular surfaces, which forms concavity compression to stabilize the joint (Halder et al., 2001). Additionally, the coordination of rotator cuff muscle contraction keeps the stress in balance in all directions, which is conducive to the stability of the shoulder joint (Lee et al., 2000; Abboud and Soslowsky, 2002). In our study, all patients had their entire humeral head and most of the rotator cuff preserved. Each insertion of the rotator cuff around the anatomical neck was marked with suture traction before the osteotomy and was sutured *in situ* after prosthesis implantation. The supraspinatus was intact and most of the infraspinatus was preserved because the insertion position was superior to the osteotomy plane. However, part of the subscapularis and most of the teres minor were removed to ensure a curative margin. Thus, we used Marlex mesh to repair the insufficient muscles. Therefore, the stability of the glenohumeral joint was well preserved and joint tension was maintained. On the basis of the stability of the glenohumeral joint, other muscles that also influence shoulder abduction, such as the deltoid muscle and pectoralis major, were carefully sutured through the designed holes on the prosthesis surface.

Our study has some limitations. First, because of the differences in the extent of the resections and disease processes, it was difficult to formulate a comparative control group. Second, there was no biomechanical analysis included in our study. Future studies should include a finite element analysis. Third, it is possible that more complications might arise after a longer follow up, but a safe surgical margin was obtained and no local recurrence was recorded at our last follow up. Therefore, further research should be performed and a longer follow up is needed.

## 5 Conclusion

A 3D printed prosthesis with cone-like structures can successfully achieve joint-sparing reconstruction of proximal humeral tumorous defects. The prosthesis was designed to improve initial stability and promote osteointegration, which ensured good survival. A surgical technique that considers shoulder joint integrity and passive tension balance results in favorable function.

## Data Availability

The original contributions presented in the study are included in the article/Supplementary Material, further inquiries can be directed to the corresponding authors.

## Supplementary material

SUPPLEMENTARY VIDEO S1 Circumduction movement in the sagittal plane of a patient.

SUPPLEMENTARY VIDEO S2 Dynamic x-ray of the shoulder joint movement of a patient.

## Abbreviations

3D, Three dimensional; 3D-CT, Three-dimensional computed tomography; MRI, Magnetic resonance imaging; SPECT, Single-photon emission computed tomography; PET/CT, Positron emission tomography/computerized tomography; STL, Stereolithography; T-SMART, Tomosynthesis-Shimadzu Metal Artefact Reduction Technology; MSTS, Musculoskeletal Tumor Society.

## References

[B1] Abboud J. A. Soslowsky L. J. (2002). Interplay of the static and dynamic restraints in glenohumeral instability. Clin. Orthop. Relat. Res. 400, 48–57. 10.1097/00003086-200207000-00007 12072745

[B2] Abudu A. Carter S. R. Grimer R. J. (1996). The outcome and functional results of diaphyseal endoprostheses after tumour excision. J. Bone Jt. Surg. Br. 78 (4), 652–657. 10.1302/0301-620x.78b4.0780652 8682837

[B3] Arndt C. A. Rose P. S. Folpe A. L. Laack N. N. (2012). Common musculoskeletal tumors of childhood and adolescence. Mayo Clin. Proc. 87 (5), 475–487. 10.1016/j.mayocp.2012.01.015 22560526 PMC3538469

[B4] Ayoub K. S. Fiorenza F. Grimer R. J. Tillman R. M. Carter S. R. (1999). Extensible endoprostheses of the humerus after resection of bone tumours. J. Bone Jt. Surg. Br. 81 (3), 495–500. 10.1302/0301-620x.81b3.0810495 10872374

[B5] Batta V. Coathup M. J. Parratt M. T. Pollock R. C. Aston W. J. Cannon S. R. (2014). Uncemented, custom-made, hydroxyapatite-coated collared distal femoral endoprostheses: Up to 18 years' follow-up. Bone Jt. J. 96-B (2), 263–269. 10.1302/0301-620x.96b2.32091 24493195

[B6] Benevenia J. Kirchner R. Patterson F. Beebe K. Wirtz D. C. Rivero S. (2016). Outcomes of a modular intercalary endoprosthesis as treatment for segmental defects of the femur, tibia, and humerus. Clin. Orthop. Relat. Res. 474 (2), 539–548. 10.1007/s11999-015-4588-z 26475032 PMC4709281

[B7] Bielack S. S. Kempf-Bielack B. Delling G. Exner G. U. Flege S. Helmke K. (2002). Prognostic factors in high-grade osteosarcoma of the extremities or trunk: An analysis of 1, 702 patients treated on neoadjuvant cooperative osteosarcoma study group protocols. J. Clin. Oncol. 20 (3), 776–790. 10.1200/jco.2002.20.3.776 11821461

[B8] Black A. W. Szabo R. M. Titelman R. M. (2007). Treatment of malignant tumors of the proximal humerus with allograft-prosthesis composite reconstruction. J. Shoulder Elb. Surg. 16 (5), 525–533. 10.1016/j.jse.2006.12.006 17560808

[B9] Ceruso M. Falcone C. Innocenti M. Delcroix L. Capanna R. Manfrini M. (2001). Skeletal reconstruction with a free vascularized fibula graft associated to bone allograft after resection of malignant bone tumor of limbs. Handchir Mikrochir Plast. Chir. 33 (4), 277–282. 10.1055/s-2001-16597 11518991

[B10] Chauhan V. S. Vaish A. Vaishya R. (2019). Reverse shoulder arthroplasty after failed megaprosthesis for osteosarcoma of the proximal humerus: A case report and review of literature. J. Clin. Orthop. Trauma 10 (3), 526–530. 10.1016/j.jcot.2019.03.015 31061583 PMC6491923

[B11] Damron T. A. Leerapun T. Hugate R. R. Shives T. C. Sim F. H. (2008). Does the second-generation intercalary humeral spacer improve on the first? Clin. Orthop. Relat. Res. 466 (6), 1309–1317. 10.1007/s11999-008-0246-z 18421535 PMC2384029

[B12] Enneking W. F. (1986). A system of staging musculoskeletal neoplasms. Clin. Orthop. Relat. Res. 204 (204), 9–24. 10.1097/00003086-198603000-00003 3456859

[B13] Gupta S. Kafchinski L. A. Gundle K. R. Saidi K. Griffin A. M. Wunder J. S. (2017). Intercalary allograft augmented with intramedullary cement and plate fixation is a reliable solution after resection of a diaphyseal tumour. Bone Jt. J. 99-B (7), 973–978. 10.1302/0301-620x.99b7.bjj-2016-0996 28663406

[B14] Halder A. M. Kuhl S. G. Zobitz M. E. Larson D. An K. N. (2001). Effects of the glenoid labrum and glenohumeral abduction on stability of the shoulder joint through concavity-compression: An *in vitro* study. J. Bone Jt. Surg. Am. 83 (7), 1062–1069. 10.2106/00004623-200107000-00013 11451977

[B15] Hara D. Nakashima Y. Sato T. Hirata M. Kanazawa M. Kohno Y. (2016). Bone bonding strength of diamond-structured porous titanium-alloy implants manufactured using the electron beam-melting technique. Mater Sci. Eng. C Mater Biol. Appl. 59, 1047–1052. 10.1016/j.msec.2015.11.025 26652463

[B16] Hardes J. Henrichs M. P. Gosheger G. Gebert C. Holl S. Dieckmann R. (2013). Endoprosthetic replacement after extra-articular resection of bone and soft-tissue tumours around the knee. Bone Jt. J. 95-B (10), 1425–1431. 10.1302/0301-620x.95b10.31740 24078544

[B17] Karageorgiou V. Kaplan D. (2005). Porosity of 3D biomaterial scaffolds and osteogenesis. Biomaterials 26 (27), 5474–5491. 10.1016/j.biomaterials.2005.02.002 15860204

[B18] King J. J. Nystrom L. M. Reimer N. B. Gibbs C. P. Jr. Scarborough M. T. Wright T. W. (2016). Allograft-prosthetic composite reverse total shoulder arthroplasty for reconstruction of proximal humerus tumor resections. J. Shoulder Elb. Surg. 25 (1), 45–54. 10.1016/j.jse.2015.06.021 26256013

[B19] Kumta S. M. Chow T. C. Griffith J. Li C. K. Kew J. Leung P. C. (1999). Classifying the location of osteosarcoma with reference to the epiphyseal plate helps determine the optimal skeletal resection in limb salvage procedures. Arch. Orthop. Trauma Surg. 119 (5-6), 327–331. 10.1007/s004020050420 10447633

[B20] Lee S. B. Kim K. J. O'Driscoll S. W. Morrey B. F. An K. N. (2000). Dynamic glenohumeral stability provided by the rotator cuff muscles in the mid-range and end-range of motion. A study in cadavera. J. Bone Jt. Surg. Am. 82 (6), 849–857. 10.2106/00004623-200006000-00012 10859105

[B21] Li J. Wang Z. Guo Z. Wu Y. Chen G. Pei G. (2012). Precise resection and biological reconstruction for patients with bone sarcomas in the proximal humerus. J. Reconstr. Microsurg 28 (6), 419–425. 10.1055/s-0032-1315766 22711209

[B22] Liu T. Zhang Q. Guo X. Zhang X. Li Z. Li X. (2014). Treatment and outcome of malignant bone tumors of the proximal humerus: Biological versus endoprosthetic reconstruction. BMC Musculoskelet. Disord. 15, 69. 10.1186/1471-2474-15-69 24607200 PMC3975708

[B23] Lu M. Li Y. Luo Y. Zhang W. Zhou Y. Tu C. (2018). Uncemented three-dimensional-printed prosthetic reconstruction for massive bone defects of the proximal tibia. World J. Surg. Oncol. 16 (1), 47. 10.1186/s12957-018-1333-6 29510728 PMC5840814

[B24] Maclean S. Malik S. S. Evans S. Gregory J. Jeys L. (2017). Reverse shoulder endoprosthesis for pathologic lesions of the proximal humerus: A minimum 3-year follow-up. J. Shoulder Elb. Surg. 26 (11), 1990–1994. 10.1016/j.jse.2017.04.005 28684229

[B25] McGrath A. Sewell M. D. Hanna S. A. Pollock R. C. Skinner J. A. Cannon S. R. (2011). Custom endoprosthetic reconstruction for malignant bone disease in the humeral diaphysis. Acta Orthop. Belg 77 (2), 171–179. 21667728

[B26] Nota S. Teunis T. Kortlever J. Ferrone M. Ready J. Gebhardt M. (2018). Functional outcomes and complications after oncologic reconstruction of the proximal humerus. J. Am. Acad. Orthop. Surg. 26 (11), 403–409. 10.5435/jaaos-d-16-00551 29762195

[B27] Palmquist A. Snis A. Emanuelsson L. Browne M. Thomsen P. (2013). Long-term biocompatibility and osseointegration of electron beam melted, free-form-fabricated solid and porous titanium alloy: Experimental studies in sheep. J. Biomater. Appl. 27 (8), 1003–1016. 10.1177/0885328211431857 22207608

[B28] Panagopoulos G. N. Mavrogenis A. F. Mauffrey C. Lesensky J. Angelini A. Megaloikonomos P. D. (2017). Intercalary reconstructions after bone tumor resections: A review of treatments. Eur. J. Orthop. Surg. Traumatol. 27 (6), 737–746. 10.1007/s00590-017-1985-x 28585185

[B29] Pilge H. Ruppert M. Bittersohl B. Westhoff B. Krauspe R. (2018). Lengthening of newly formed humerus after autologous fibula graft transplantation following intercalary tumor resection. J. Pediatr. Orthop. B 27 (4), 322–325. 10.1097/bpb.0000000000000464 28489628

[B30] Pilliar R. M. Lee J. M. Maniatopoulos C. (1986). Observations on the effect of movement on bone ingrowth into porous-surfaced implants. Clin. Orthop. Relat. Res. 208 (208), 108–113. 10.1097/00003086-198607000-00023 3720113

[B31] Potter B. K. Adams S. C. Pitcher J. D. Jr. (2009). Proximal humerus reconstructions for tumors. Clin. Orthop. Relat. Res. 467 (4), 1035–1041. 10.1007/s11999-008-0531-x 18820983 PMC2650043

[B32] Rafalla A. A. Abdullah E. S. A. (2017). Endoprosthetic replacement versus cement spacer in reconstruction of proximal humerus after tumor resection: Cost and benefits. J. Orthop. Surg. Hong. Kong) 25 (2), 230949901771393. 10.1177/2309499017713937 28625098

[B33] Ruggieri P. Mavrogenis A. F. Guerra G. Mercuri M. (2011). Preliminary results after reconstruction of bony defects of the proximal humerus with an allograft-resurfacing composite. J. Bone Jt. Surg. Br. 93 (8), 1098–1103. 10.1302/0301-620x.93b8.26011 21768636

[B34] Sewell M. D. Hanna S. A. McGrath A. Aston W. J. Blunn G. W. Pollock R. C. (2011). Intercalary diaphyseal endoprosthetic reconstruction for malignant tibial bone tumours. J. Bone Jt. Surg. Br. 93 (8), 1111–1117. 10.1302/0301-620x.93b8.25750 21768638

[B35] Shah F. A. Omar O. Suska F. Snis A. Matic A. Emanuelsson L. (2016). Long-term osseointegration of 3D printed CoCr constructs with an interconnected open-pore architecture prepared by electron beam melting. Acta Biomater. 36, 296–309. 10.1016/j.actbio.2016.03.033 27000553

[B36] Wang C. Liu D. Xie Q. Liu J. Deng S. Gong K. (2019). A 3D printed porous titanium alloy rod with diamond crystal lattice for treatment of the early-stage femoral head osteonecrosis in sheep. Int. J. Med. Sci. 16 (3), 486–493. 10.7150/ijms.30832 30911283 PMC6428983

[B37] Wieser K. Modaressi K. Seeli F. Fuchs B. (2013). Autologous double-barrel vascularized fibula bone graft for arthrodesis of the shoulder after tumor resection. Arch. Orthop. Trauma Surg. 133 (9), 1219–1224. 10.1007/s00402-013-1795-5 23793479

[B38] Wittig J. C. Bickels J. Kellar-Graney K. L. Kim F. H. Malawer M. M. (2002). Osteosarcoma of the proximal humerus: Long-term results with limb-sparing surgery. Clin. Orthop. Relat. Res. 397, 156–176. 10.1097/00003086-200204000-00021 11953608

[B39] Yoshida Y. Osaka S. Tokuhashi Y. (2010). Analysis of limb function after various reconstruction methods according to tumor location following resection of pediatric malignant bone tumors. World J. Surg. Oncol. 8, 39. 10.1186/1477-7819-8-39 20482815 PMC2881919

[B40] Zekry K. M. Yamamoto N. Hayashi K. Takeuchi A. Alkhooly A. Z. A. Abd-Elfattah A. S. (2019). Reconstruction of intercalary bone defect after resection of malignant bone tumor. J. Orthop. Surg. Hong. Kong) 27 (1), 230949901983297. 10.1177/2309499019832970 30879390

[B41] Zhao D. Tang F. Min L. Lu M. Wang J. Zhang Y. (2020). <p&gt;Intercalary reconstruction of the “ultra-critical sized bone defect” by 3D-printed porous prosthesis after resection of tibial malignant tumor</p&gt;. Cancer Manag. Res. 12, 2503–2512. 10.2147/cmar.s245949 32308487 PMC7152541

[B42] Zheng K. Yu X. C. Hu Y. C. Shao Z. W. Xu M. Wang B. C. (2019). Outcome of segmental prosthesis reconstruction for diaphyseal bone tumors: A multi-center retrospective study. BMC Cancer 19 (1), 638. 10.1186/s12885-019-5865-0 31253134 PMC6599373

